# A Novel Compound Heterozygous Variant in the *ABHD12* Gene Cause PHARC Syndrome in a Chinese Family: The Proband Presenting New Genotype and Phenotype

**DOI:** 10.1002/mgg3.70055

**Published:** 2025-02-05

**Authors:** Meijiao Ma, Jinhai Ma, Yuanyuan Lian, Xueli Wu, Wenming Wang, Weining Rong, Xunlun Sheng

**Affiliations:** ^1^ Gansu Aier Ophthalmology and Optometry Hospital Lanzhou China; ^2^ Lanzhou Brigtt Eye Hospital Lanzhou Lanzhou China; ^3^ Ningxia Eye Hospital, People's Hospital of Ningxia Hui Autonomous Region Third Clinical Medical College of Ningxia Medical University Yinchuan China

**Keywords:** *ABHD12*, Chinese, new genotype‐phenotype, PHARC

## Abstract

**Background:**

PHARC syndrome, a rare autosomal recessive neurodegenerative disorder caused by mutations in the *ABHD12* gene, is characterized by demyelinating polyneuropathy, hearing loss, ataxia, retinitis pigmentosa (RP), and early‐onset cataracts. If patients are first diagnosed in the ophthalmology department, they are easily misdiagnosed as having RP or Usher syndrome. This study aimed to identify the genetic etiology and determine the clinical diagnosis of a Chinese family with suspected PHARC syndrome.

**Method:**

Comprehensive ophthalmic examinations and systemic evaluations were conducted to confirm the phenotype. The genotype was identified through Whole Exome Sequencing (WES), and the current literature was reviewed understand better manifestations of PHARC syndrome related to pathogenic variants.

**Results:**

The principal symptoms of the proband comprised profound sensorineural hearing loss since childhood, severe visual impairment, congenital cataracts, cone‐rod dystrophy, and ataxia. WES revealed that the proband carried a compound heterozygous variant in the *ABHD12* gene: M1, a known nonsense variation c.477G > A (p.Trp159Ter); and M2, a novel copy number variant with a deletion of approximately 18.10 Kbp in chromosome 20p11.21 (seq[GRCh38]del(20) (p11.21)chr20:g. 25302218_25320318del), covering exons 4–12 of the *ABHD12* gene. The literature review indicated that there were 65 patients with PHARC from 30 different families. All clinical information of the described patients with PHARC syndrome and all known mutations associated with the disease to date were compiled.

**Conclusion:**

This study expands the spectrum of pathogenic variants and phenotype for PHARC syndrome and suggests genetic testing is necessary for a definitive diagnosis of PHARC syndrome.

## Graphical abstract

The principal symptoms of the proband comprised profound sensorineural hearing loss since childhood, severe visual impairment, congenital cataracts, cone‐rod dystrophy, and ataxia. Whole Exome Sequencing (WES) revealed that the proband carried a compound heterozygous variant in the ABHD12 gene: M1, a known nonsense variation c.477G>A (p.Trp159Ter); and M2, a novel copy number variant with a deletion of approximately 18.10 Kbp in chromosome 20p11.21 (seq[GRCh38]del(20)(p11.21)chr20:g. 25302218_25320318del), covering exons 4‐12 of the ABHD12 gene. The literature review indicated that there were 65 patients with PHARC from 30 different families. All clinical information of the described patients with PHARC syndrome and all known mutations associated with the disease to date were compiled.

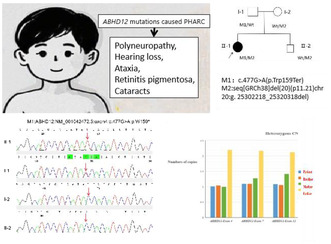

## 1 Introduction

PHARC syndrome (Polyneuropathy, Hearing loss, Ataxia, Retinitis pigmentosa (RP), and Cataracts) (MIM# 612674) is an autosomal recessive neurodegenerative disorder caused by mutations in the *ABHD12* gene. It is clinically characterized by ataxia, demyelinating neuropathy, peripheral neuropathy, hearing loss, RP, and cataract. To date, 65 cases of PHARC syndrome have been reported worldwide (Demir et al. 2024; Hernández‐Emanuelli, Emanuelli, and Izquierdo 2024; Daneshi et al. 2023; Long et al. 2024; Harutyunyan, Callaerts, and Vermeer 2024), and no population‐based incidence or prevalence data are available. The disease was first described in Norwegian patients in 2009 (Fiskerstrand et al. 2009), and then Fiskerstrand et al. identified *ABHD12* as the causative gene of PHARC syndrome in 2010 (Fiskerstrand et al. 2010). Due to the diversity of clinical phenotypes among patients, it is often misdiagnosed as other degenerative neuropathies with similar symptoms, such as mitochondrial disease or Refsum's disease (Fiskerstrand et al. 2009). If a patient is first diagnosed in the ophthalmology department, they are susceptible to misdiagnosis as RP and Usher's syndrome (Fiskerstrand et al. 2009, 2010; Lerat et al. 2017; Frasquet et al. 2018). With the continuous maturation of genetic screening technology, especially the development of NGS technology, genetic testing of such patients has revealed that the *ABHD12* gene is the only one that has been identified to be associated with the pathogenesis of PHARC syndrome. Most of the reported cases are from Europe, and a few have been reported in Asians.

**FIGURE 1:**
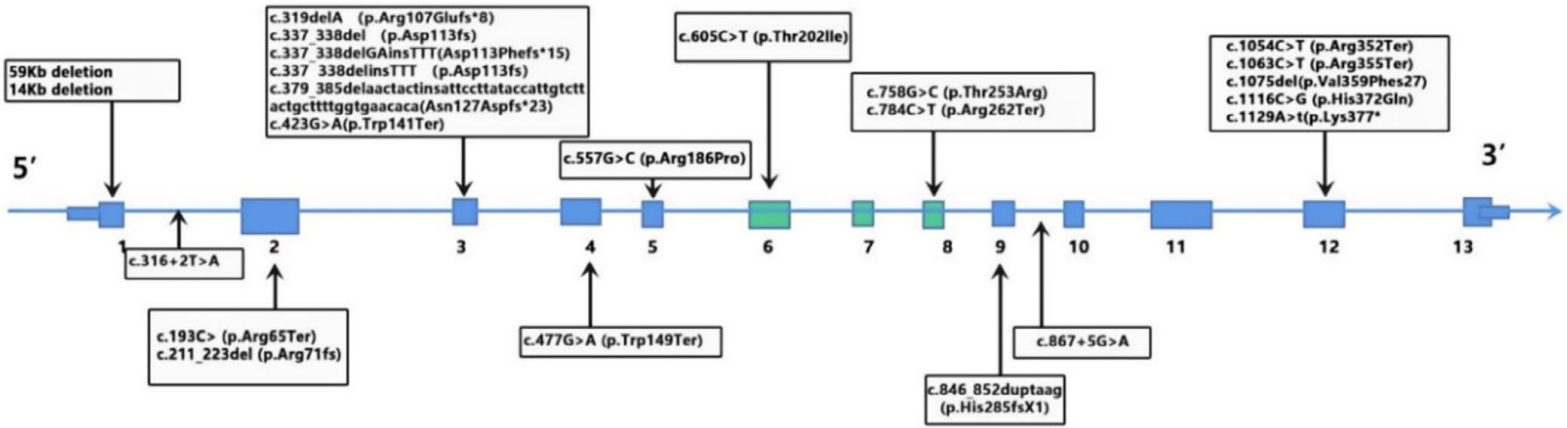
Exonic organization of ABHD12 and published mutations in PHARC.

The *ABHD12* gene spans approximately 170 kb on chromosome 20p11 and consists of 13 exons that form a coding region of 1215 nucleotides (Kind and Kursula 2019). As of July 7, 2024, 49 pathogenic variants and 20 likely pathogenic variants of the *ABHD12* gene have been reported in the ClinVar database (http://www.ncbi.nlm.nih.gov/clinvar). The types of variants include frameshift variation, missense variation, nonsense variation, and splice site variation (Figure 1).

PHARC syndrome exhibit a high degree of clinical heterogeneity in terms of severity and progression. Patients may present with symptoms such as neurodegeneration, RP, cataract, or hearing loss as the first symptom, but the degree of severity and disease progression varies. For example, the most common frameshift variant c.337_338delGAinsTTT (P.Asp113Phefs15) was found in 17 patients, who were mainly characterized by demyelinating neuropathy, cataract, and hearing loss manifestations predominantly, with only two cases presenting with RP (Lerat et al. 2017). The frameshift variant c.846_852dupTAAGAGC (p.His285fs1) predominantly caused demyelinating neuropathy in seven patients, whereas only one patient exhibited cataracts, RP, and hearing loss (Lerat et al. 2017). In contrast, the compound heterozygous variants c.337delA (p.Arg107Glufs*8) and c.605C>T (p.Thr202Ile) led to RP and cataract, with no hearing impairment or neurological abnormalities found (Nishiguchi et al. 2014). The missense variant c.784C>T (p.Arg262Ter) was insignificant in RP in two patients from the same family (Thimm et al. 2020; Nguyen et al. 2021).

Owing to the heterogeneous nature of the clinical manifestations of PHARC syndrome and the dearth of comprehensive and standardized clinical criteria, genetic testing is frequently indispensable for a definitive diagnosis of the majority of PHARC syndrome cases. The introduction of whole exome sequencing (WES) has augmented the accuracy of diagnosing these diseases and revolutionized the process of characterizing the disease's phenotypic profile. Consequently, the phenotypic range of recognized Mendelian diseases has been expanded. Herein, we report a Chinese Han patient with PHARC syndrome who harbored a novel compound heterozygous variant detected in the *ABHD12* gene, and whose ocular clinical phenotype was cone‐rod dystrophy. This differs from previous reports where RP was the first ocular phenotype in patients with PHARC syndrome. Based on the review and analysis of the available literature, this is the third report of PHARC syndrome in Chinese people (Long et al. 2024; Thimm et al. 2020).

## 2 Data and Methods

### 2.1 General Data

A pedigree of PHARC syndrome was collected from Gansu Aier Ophthalmology and Optometry Hospital. The pedigree encompassed four members spanning two generations, with one patient being identified. Thorough inquiries were conducted regarding the family history, previous medical history, as well as marital, and childbearing history of the proband and their parents. Subsequently, a family tree was constructed.

Approval for this research project was granted by the Ethics Committee of Gansu Aier Ophthalmology and Optometry Hospital [Approval No. GSAIER2023IRB03], strictly adhering to the Declaration of Helsinki. All participants in this study volunteered and furnished their signed informed consent.

### 2.2 Methods

#### 2.2.1 Clinical Examination

All individuals in this family were recruited to undergo comprehensive ophthalmic examinations. An international standard visual acuity chart was used to examine unaided visual acuity, comprehensive optometry techniques were adopted to determine the best—corrected visual acuity, a non—contact tonometer was used to measure intraocular pressure, the slit—lamp microscope was employed to examine the anterior segment of the eye, and three—mirror contact lenses and color fundus photography were used for fundus examinations. Fundus autofluorescence was carried out to assess the lipofuscin content and distribution in the retinal pigment epithelium, optical coherence tomography (OCT) was performed to evaluate retinal morphology and macular structure, flash electroretinogram (F—ERG) was used to evaluate the retinal function, and visual field examinations were conducted to assess the extent of visual field damage in the patient. Patient's hearing tests and neurological examinations were evaluated by experienced specialists.

#### 2.2.2 Genetic Testing and Analysis

2 mL of peripheral venous blood was collected from the proband and family members. Genomic DNA was extracted by a DNA extraction kit (Qiamo Blood Mini Kit DNA), and the genomic library was constructed after passing the concentration and purity tests. The exons, adjacent splicing regions (about 20 bp) of the target gene and the full length of mitochondrial genome were captured and enriched by probe hybridization. The enriched genes were subjected to quality control and sequenced using a high‐throughput sequencer. The amount of original sequencing data captured for the target region was 1,378,891, the total number of sequencing reads was 91,926,060, and the average sequencing depth was 157.15X. The sequencing parameters captured for the target region of the tested samples were shown in Table 1. After removing the reads that did not meet the quality control requirements from the original sequencing data, the BWA software was used to compare the sequences with the hg38 human genome reference sequence provided by UCSC. The SNV and InDel variants were identified therein by GATK's HaplotypeCaller, and then through professional database and bioinformatics predication software, xhmm and clamms algorithms were used to analyze the copy number variation (CNV) in the probe coverage. Co‐segregation of family members was verified using Sanger validation. Real‐time quantitative polymerase chain reaction (RT‐qPCR) was applied to validate the detected copy number deletion (CNV) of the *ABHD12* gene. Genomic DNA was extracted from peripheral blood of family members using a Blood Genomic DNA Extraction Kit (Beijing Tiangen Biochemical Technology Co. Ltd). Primers were designed on exon 4, exon 7, exon 12 of *ABHD12* gene, meanwhile, primers were also designed on exon 9 of TGM3 gene and exon 3 of *ZBTB46* gene as upsream control and downsream control espectively (Table 1).

**TABLE 1:** Genetic testing data.

Amount of original sequencing data captured for the target region	1,378,891
Total number of sequencing reads	91,926,060
Average sequencing depth	157.15X
Average sequencing depth of target area ≥ 1X coverage	99.75%
Average sequencing depth of target area ≥ 10X coverage	99.17%
Average sequencing depth of target area ≥ 30X coverage	99.17%
Average mitochondrial genome sequencing depth	11,351.26X
Q30 Pass rate	92.04%

The RT‐qPCR reaction system was prepared according to the manufacturer's specifications (NovoStart SYBR qPCR SuperMix Plus kit) and carried out on a Roche Light Cycler II 480 Real‐Time PCR System (Roche Applied Science, Shanghai, China). Each 20 μL of the PCR mixture contained 10 μL 2 × NovoStart SYBR qPCR SuperMix Plus, 0.5 μL each primer, 1 μL gDNA (10 ng), and 8 μL ddH2O. After an initial denaturing for 1 min at 95°C, there were 40 cycles of amplification (95°C for 20 s and 60°C for 20 s), each reaction was run in triplicate. The relative copy number of *ABHD12* gene was calculated and normalized against the reference gene *GAPDH* based on 2^−ΔΔCt^ method. For autosomal chromosomes, the relative copy numbers around 2 indicated a normal copy number status, and a value around 1 indicated a loss CNV status (Table 2).

**TABLE 2:** Real‐time quantitative PCR primer information.

Primer name	Forward primer (5′‐3′)	Reverse primer (5′‐3′)	Location
SG10470‐Qpcr	TGCAGCTGAACTTCGACATG	TGTTTGCCAGTGGTGTTGTC	*TGM3*‐Exon 9
SG10462‐Qpcr	CTGGTACCTGCGTTCCCATG	TGCAGTCTGGTGGAAGAACG	*ABHD12*‐Exon 4
SG10463‐Qpcr	ATGTACACGGGGTTGTCACC	TTGGGGTGACTCAGTGGGAA	*ABHD12*‐Exon 7
SG10464‐Qpcr	CTCAGTATCCGTGGCAGCTC	CTCTATAGCATCGCCGCACC	*ABHD12*‐Exon 12
SG10465‐Qpcr	CGTGAACTTCTTCCCGCAGA	GTGTCCGTACTGCAGCTTCT	*ZBTB46*‐Exon 3
GAPDH	TACTAGCGGTTTTACGGGCG	GAACAGGAGGAGCAGAGAGCG	Autosomal chromosome
XP60	GTGACAGGAGGAACGGAAGGGTT	CTGTTTTACCTGGCTAAGGTTGTG	X chromosome

Interpretation rules for variant data were aligned with the Standards and Guidelines for Classification of Genetic Variants issued by the American College of Medical Genetics and Genomics (ACMG) (Richards et al. 2015) and incorporated a series of general recommendations and specifications successively issued by the ClinGen Sequence Variants Interpretation (SVI) Expert Group Interpretation rules for CNVs adhered to the 2019 ACMG CNV Interpretation and Reporting Guidelines (Riggs et al. 2020). The *ABHD12* WT and *ABHD12* p.W159Ter models were generated using alphafold software to validate the effect of the M1 variant on protein function.

#### 2.2.3 Literature Review

A search was conducted on PubMed using “PHARC,” “*ABHD12*,” and “polyneuropathy hearing loss ataxia RP cataracts” as the terms to search the relevant literature for analyzing the clinical manifestations and disease characteristics of PHARC syndrome caused by various variants.

## 3 Results

### 3.1 Clinical Characteristics of the Family

**FIGURE 2:**
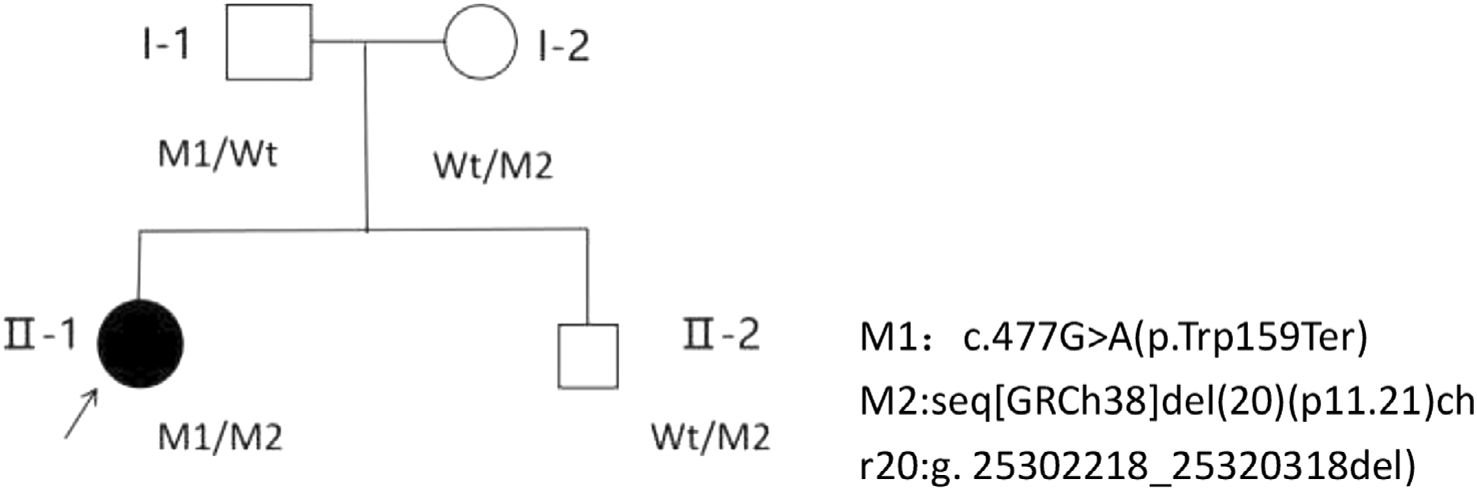
Pedigree of family.

**FIGURE 3:**
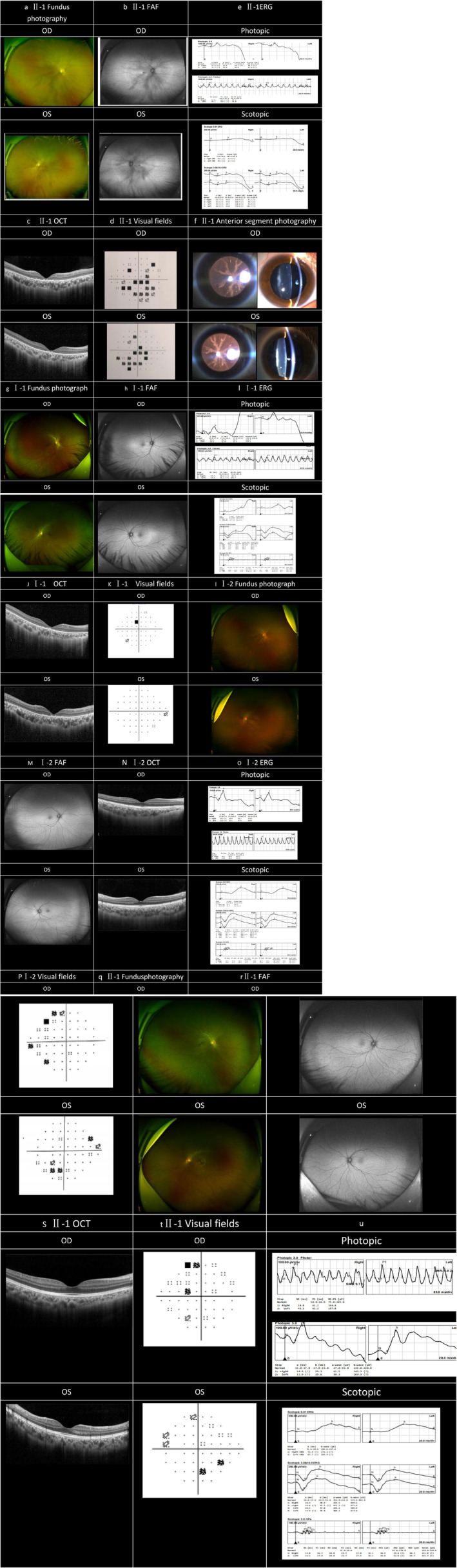
Ocular examination of proband and family member. (a) II‐1 Fundus Examination: No visible peripheral osteocyte‐like pigmentation was observed. (b) FAF: An expanded area of hypofluorescence at the macular fovea, surrounded by hyperfluorescent rings on the temporal side. (c) Optical coherence tomography (OCT): Thinning of the outer retina and interrupted continuity of the ellipsoid zone and external limiting membrane, with visible hyperreflective masses in the RPE/Bruch complex zone. (d)Visual field:Central and paracentric scotoma. (e) ERG: Severe decrease in rod and cone response. (f) Anterior Segment Photography: Coral‐like opacity noted in the lens. (j, k) In the proband's father (I‐1): Autofluorescence revealed an expanded area of hypofluorescence at the macular fovea. OCT indicated thinning of the outer retina and disruption in the ellipsoidal zone at the fovea centralis. No abnormalities in ERG or visual field were detected. (i–p) In the proband's mother (I‐2): No ophthalmic abnormalities were detected. (q–u) In the proband's brother (II‐2): No ophthalmic abnormalities were detected.

The proband, a 24‐year‐old female, had no history of consanguineous marriages within three generations of her family, and denied any family history of hereditary diseases (Figure 2). She had poor binocular vision, an unstable walking gait, and was prone to falling since childhood. At the age of 16 years, she underwent cochlear implantation for bilateral profound sensorineural hearing loss. She visited the ophthalmology department due to a rapid deterioration in vision in both eyes over the past 2 years. Eye examination revealed normal color vision; best corrected visual acuity registered at 0.1 (−2.25/−2.50170) in the right eye and 0.1 (−2.25/−1.75170) in the left eye. Intraocular pressure measured 16.7 mmHg in the right eye and 15.3 mmHg in the left eye (Table 3). Both eyes exhibited transparent corneas, normal‐depth anterior chambers, clear‐textured irises, round pupils approximately 2.5 mm in diameter, light reflex sensitivity, and coral‐like lens opacities (Figure 3f). Vitreous transparency and no obvious abnormalities under three‐mirror contact lens. There were no visible peripheral osteocyte‐like pigmentation by fundus examination (Figure 3a). Autofluorescence (FAF) revealed an expanded area of hypofluorescence at the macular fovea, surrounded by hyperfluorescent rings on the temporal side (Figure 3b). OCT demonstrated thinning of the outer retina and disruption in the continuity of the ellipsoid zone and external limiting membrane, with hyperreflective masses evident in the RPE/Bruch complex zone (Figure 3c). The visual field displayed central and paracentric scotoma (Figure 3d), accompanied by a marked reduction in rod and cone response in the ERG (Figure 3e). The clinical diagnosis of cone‐rod dystrophy and congenital cataract was established based on her ocular findings. In the proband's father (I‐1), autofluorescence revealed an expanded area of hypofluorescence at the macular fovea; OCT demonstrated thinning of the outer retina and disruption of the ellipsoidal zone in the fovea centralis; no abnormalities were observed in the ERG and visual field (Figure 3j,k). No ophthalmic abnormalities were detected in the proband's mother and younger brother (Figure 3i–u). Neurological examination of the proband revealed an abnormal finger‐nose test and a positive Romberg test, indicative of sensory ataxia. She experienced decreased superficial sensation on the left side compared to the right, alongside foot drop; however, muscle strength, and reflexes were normal. The proband could not undergo MRI due to previous cochlear implantation, and cranial CT revealed no evident signs of cerebellar atrophy. Other family members showed no neurological manifestations.

**TABLE 3:** Eye clinical findings.

Samples	Age	BCVA		IOP mmHg	
		OD	OS	OD	OS
II‐1	24	−2.25DS:−2.50DCDC*170°:0.1	−2.25DS:−1.75 DC*170°:0.1	16	15
I‐1	46	−2.75DS:−0.50DCDC*22°:1.0	−2.50DS:−0.25 DC*180°:1.0	14	14
I‐2	46	−0.25DS:−0.50DCDC*174°:1.0	−0.50DS:+0.00 DC*0°:1.0	12	10
II‐2	19	−2.75D°:−1.25DCDC*2°:1.0	−3.00DS:−1.00 DC*178°:1.2	14	12

### 3.2 Whole Exome Sequencing

WES of the proband's DNA revealed two heterozygous variants in the *ABHD12* gene. M1 was a nonsense variant, c.477G > A:p.W159Ter, located in exon 4; this change from base G to A at cDNA position 477 led to the 159th codon altering from tryptophan to a stop codon. M2 involved a copy number loss in the region chr20:25302218–25320318 affecting exons 4–12 of the *ABHD12* gene, included in the OMIM as a known pathogenic gene. Owing to the restricted capacity of exon sequencing in detecting CNVs, qPCR was carried out on the subjects and their related family members for the purpose of further testing and validation.

### 3.3 Sanger Sequencing Validation, qPCR Validation, and Familial Co‐Segregation Analysis

**FIGURE 4:**
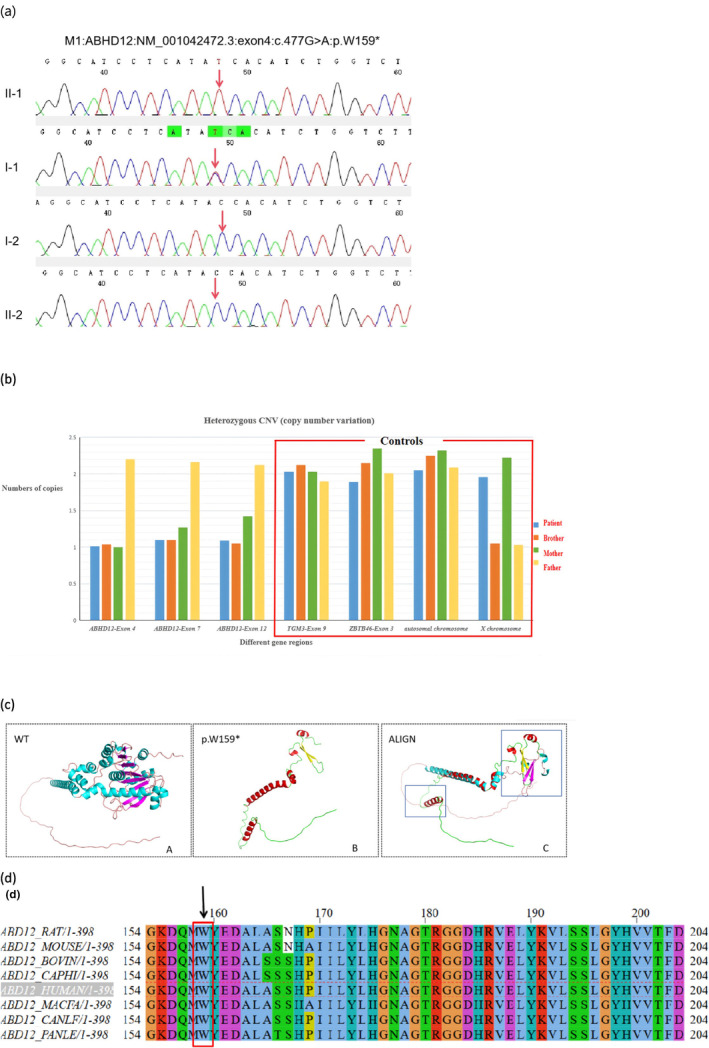
M1 and M2 mutations verification and analysis. (a) Sequence Chromatograms of M1 Mutations: Both the proband and the father carried the M1 variant in the ABHD12 gene (c.477G>A:P.W159*), which was not detected in the mother and younger brother. (b) qPCR Validation Results of the Target Gene Region in the Proband's Family: QPCR was performed on all family members, confirming the presence of the M2 variant (seq[GRCh38] del(20) (p11.21) chr20:G. 25302218_25320318del) in the proband, younger brother, and mother. (c) M1 Nonsense Variant Producing Truncated Protein and Affecting Protein Function. The *ABHD12* gene, composed of 398 amino acids, features a three‐dimensional structure with 14 α‐helices, 8 β‐folds, and 18 loop regions. The p.W159* mutation results in the termination of gene translation at tryptophan 159, reducing the protein to 5 α‐helices, 2 β‐folds, and 8 irregular loops. A: Wild type (purple: β‐fold; blue: α‐helix; pink: Irregular loop zone); B: Mutant (red: α‐helix, yellow: β‐fold, green: Irregular loop region); C: Folded structure of the protein, showing a transition to an α‐helix structure in the wild‐type N‐terminal loop region post‐mutation. This loss of components leads to significant changes in protein function and structure. The blue box highlights a major structural change post‐absence. (d) The Homology of Amino Acid Sequences Between Human *ABHD12* and Other Species: The amino acid at position 159 is highly conserved among species, and the mutated residue at position 159 is marked with a black arrow.

Validation of Sanger sequencing for all family members revealed that both the proband and the father carried the M1 variant in the *ABHD12* gene (c.477G > A:p.W159*), not detected in the mother and younger brother, as previously reported (Figure 4a) (Nguyen et al. 2021). qPCR was conducted on all family members, confirming the presence of the M2 variant (seq[GRCh38] del(20) (p11.21) chr20:g.25302218_25320318del) in the proband, younger brother, and mother. According to Mendel's laws of inheritance, the M1 variant (c.477G > A:p.W159*) originated from the father, while the M2 variant (copy number loss in the region chr20:25302218–25320318) originated from the mother (Figure 4b). The compound heterozygous variant co‐segregated with the phenotype, conforming to an autosomal recessive pattern of inheritance.

### 3.4 Analysis of Pathogenicity

After WES was performed, compound heterozygous variants were detected in the *ABHD12* gene of the proband. M1 nonsense variant c.477G > A(p.Trp159Ter) resulted in the 159th codon changing from encoded tryptophan to terminating codon, this amino acid position exhibits high conservation across various species, thus producing truncated protein and affecting the function of the protein thereby and indicating Pathogenic Very Strong (PVS1) (Figure 4c,d). This variant was a rare variant, and a frequency of 0 in the general population of East Asia in the gnomAD database indicated Pathogenic Moderate (PM2). The Sanger sequencing showed that M1 variant was inherited from the father, and was not detected in the mother and younger brother, and the familial co‐segregation demonstrated Pathogenic Supporting (PP1). Such variant was classified as pathogenic (PVS1+ PM2 + PP1) according to the ACMG guideline. M2 was a deletion of approximately 18.10 Kbp in chromosome 20p11.21 (seq[GRCh38]del(20) (p11.21)chr20:g. 25302218_25320318del). qPCR validation of copy number loss in the region chr20:25302218–25320318 showed that the heterozygous variants in this region were detected from the proband, the younger brother and mother of the proband. Such variant was the null variant, which could cause the protein to become shorter in length and further affect the structure of the protein. Its pathogenic mechanism was loss of function (LOF), indicating PVS1. This variant was a rare variant and not found in the database of genomic variants normal population database, indicating Pathogenic Moderate (PM2). Such variant was likely located in the transposition of the pathogenic variant M1, indicating Pathogenic Moderate (PM3). Therefore, this variant was classified as pathogenic (PVS1 + PM2 + PM3) as assessed by the principles and guidelines for interpretation of sequence variation.

### 3.5 Previously Reported *ABHD12* Gene Variants and Associated Clinical Phenotypes

Totally 32 paper were found, and selected that reported both genetic test results and clinical characteristics. Finally, 17 articles were cited. The literature review showed that about 64 cases (Demir et al. 2024; Hernández‐Emanuelli, Emanuelli, and Izquierdo 2024; Daneshi et al. 2023; Long et al. 2024; Harutyunyan, Callaerts, and Vermeer 2024) worldwide with significant features of PHARC syndrome had been reported. All clinical information of the described patients with PHARC syndrome and all known mutations associated with the disease to date was compiled and is presented in Table 4.

**TABLE 4:** The manifestations of patients with ABHD12 variations in literature and the patient in this study.

Reference	Patients number	*ABHD12* Nucleotide change	Sensory and Motor Neuropathy	Hearing Loss	Ataxia	Retinitis Pigmentosa	Cataract
(Long et al. 2024)	2	c.690G>A	0	2	1	1	1
(Wawrocka et al. 2024)	1	c.874 C>T/c.205_206del	0	1	0	1	0
(Hernández‐Emanuelli, Emanuelli, and Izquierdo 2024)	1	c.1063C>T, p.(Arg355Ter)	1	1	1	1	0
(Demir et al. 2024)	1	Exon 1 deletion	1	1	0	1	1
(Daneshi et al. 2023)	1	c.871del (p.Tyr291IlefsTer28)	1	1	0	1	1
(Nguyen et al. 2021)	2	c.601dup, p.(Val201GlyfsTer4)	2	2	2	2	2
(Dias Bastos et al. 2021)	1	c.337_338delGAinsTTT/c.1075del	1	1	1	1	1
(Thimm et al. 2020)	1	c.337_338delGAinsTTT/c.337_338delGAlinsTTT	1	1	1	1	1
(Li et al. 2019)	3	c.337_338delGAinsTTT/c.423‐1_425del	3	2	3	3	3
(Frasquet et al. 2018)	1	c.477G>A/c.557G>C	1	1	1	1	1
(Lerat et al. 2017)	1	c.337_338delGAinsTTT/c.337_338delGAlinsTTT	0	1	1	1	1
(Chen et al. 2013)	1	c.784C>T/c.867 + 5G>A	1	1	1	1	0
(Eisenberger et al. 2012)	2	p.Arg65X	1	2	1	2	2
(Fiskerstrand et al. 2010)	8	c.337_338delGAinsTTT	8	8	3	6	8
	3	14 Kb deletion including exon 1	3	3	3	2	3
	1	c.1054C>T [p.Arg352X]	1	1	1	1	1
	7	c.846_852dupTAAGAGC [p.His285fsX1]	7	4	6	3	1
(Tingaud‐Sequeira et al. 2017)	1	c.758C>G	1	1	1	1	1
(Yoshimura et al. 2015)	4	c.316 + 2 T>	1	4	1	3	3
(Nishiguchi et al. 2014)	4	c.319delA/c.605c>T	0	2	0	4	4
	1	c.1116C>G	1	1	0	0	1
	1	c.447 g>A/c.557 g>C	0	0	1	0	0
Our study	1	C.477G>A (p.Trp159Ter)	1	1	1	1	1

## 4 Discussion

The *ABHD12* gene encodes a protein comprising 398 amino acids and this protein's primary structure is fully conserved between rodents and humans, while its tertiary structure consists of a transmembrane protein featuring 14 α‐helices, 8 β‐sheets, and 18 loop regions (Thimm et al. 2020). The active sites are positioned on the extracellular surface at the 246th, 333rd, and 372nd amino acids, respectively (Thimm et al. 2020). The encoded enzyme, ABHD12 protease, belongs to the metabolic serine hydrolases family. It is expressed in multiple tissues and organs, exhibiting peak expression levels in the brain (Blankman, Simon, and Cravatt 2007). The main physiological functions are two‐fold: Firstly, it catalyzes the hydrolysis of 2‐arachidonic glycerol (2‐AG), a critical endocannabinoid lipid transmitter that interacts with the cannabinoid receptors CB1 and CB2. These endocannabinoids partake in numerous physiological processes such as neurotransmission, mood regulation, appetite, pain perception, addiction, and inflammation; Second, as phosphatidylserine (LPS) synthesized by lipase hydrolysis of *ABHD16A*, the disruption of *ABHD12* in mice leads to dysregulation of LPS in the cerebellum, which induces sustained stimulation of Purkinje neurons, resulting in dysregulation of cerebellar activity, activation of microglial cells, and elevated levels of inflammation. Therefore, *ABHD12* variation can lead to affected physiological behaviors involving the endocannabinoid system and cause abnormalities in lipid metabolism in vivo triggering behavioral deficits (Shin et al. 2019; Joshi et al. 2018). Studies on a PHARC syndrome model, created by knocking out the *ABHD12* gene in mice, revealed that as *ABHD12*−/− mice aged, they developed hearing impairment and abnormal motor behavior. Additionally, early stages (2–6 months) showed increased lysophosphatidylserine lipids, and subsequent activation of microglia led to neuroinflammatory responses, as well as further hearing and motor defects (Kamat et al. 2015). Zebrafish with *ABHD12* gene dysfunction exhibited progressive ataxia, motor skill disorder, retinal dysfunction, cataract, and decreased hair cells in the inner ear. These symptoms were alleviated by introducing wild‐type *ABHD12* mRNA, whereas introducing mutant *ABHD12* mRNA had no such effect (Tingaud‐Sequeira et al. 2017).

Hearing loss represents the most prevalent symptom of PHARC syndrome. In the majority of cases, it emerges prior to the age of 15 years and typically progresses to deafness as the disease advances. The hearing condition can be ameliorated through cochlear implantation. Our patient, who had profound hearing loss, underwent cochlear implantation at the age of 15 years and is now leading a normal life without any hindrance, thereby demonstrating that patients with PHARC syndrome can enhance their hearing via cochlear implantation. Nevertheless, the precise localization and role of *ABHD12* within the inner ear remain elusive and may involve the distal modulation of microglial cells in the inner ear by the central nervous system (Minamihata et al. 2022). One of the initial and common ocular symptoms is cataracts, which typically manifest around the age of 20 (Nguyen et al. 2021). Our patient was diagnosed with cataracts in both eyes at the age of 24. The onset of cataracts might be associated with retinal degeneration, as the latter elevates the pro‐inflammatory cytokines and catalytic factors within the vitreous body, thereby altering the lens homeostasis and leading to lens opacity. Retinal pigmentation (RP) is another highly prevalent ocular symptom, typically occurring between the ages of 20 and 30. Some patients may exhibit classic RP features, such as attenuated retinal blood vessels, intraretinal pigmentation, waxy pallor of the optic disc, severely constricted visual fields, and significantly reduced rod and cone responses in ERG. Some patients may lack intraretinal pigmentation; however, the presence of constricted visual fields and a significantly reduced rod‐cone response in ERG indicates a severe decline in rod and cone function. Nguyen XT (Nguyen et al. 2021) reported on 15 patients with PHARC syndrome: all patients exhibited signs of retinal degeneration on fundus examination, and only seven cases (47%) displayed typical retinal osteoblast‐like pigmentation; the best corrected visual acuity in patients with pigmentation was lower than that in patients without. Most patients exhibited hypofluorescence in the macular region during autofluorescence examination; spectral‐domain optical coherence tomography (SD‐OCT) revealed degeneration of the outer retina, featuring macular dystrophy and early vision loss in the initial stages. In four patients, visual acuity was relatively preserved, and OCT revealed preserved structures in the ELM/EZ region. Therefore, the progression of the disease can be evaluated by means of fundus multimodal imaging combined with either ERG or visual field. In this study, no intraretinal pigmentation was observed in the fundus of the proband, and neither the visual field nor other fundus examinations supported the diagnosis of RP. The proband initially presented with decreased visual acuity and reported no history of night blindness. Clinical examination revealed severely impaired central vision, and the visual field showed central scotoma and paracentric scotoma. The autofluorescence indicated hypofluorescence in the macular region, and the OCT revealed the structural destruction of the external limiting membrane/ellipsoid (ELM/EZ) region. Clinical examination confirmed the diagnosis of cone‐rod dystrophy (CORD). CORD initially affects the cones and subsequently involves the rods. Unlike RP, CORD manifests initially with cone involvement in the macular region, while RP, as a rod‐cone dystrophy (RCD), originates from the peripheral retina with a pathogenesis that is the exact opposite. RP is characterized by early night vision impairment and peripheral vision loss, while CORD presents with early central vision impairment in the disease course.

The molecular mechanism by which *ABHD12* gene mutations lead to cataract and RP remains unclear. Knockdown of the *ABHD12* gene in mice led to neurological and auditory abnormalities, but did not result in retinal degeneration or lens opacity (Blankman et al. 2013). This suggests limited expression in the retina and lens, potentially attributable to species differences. *ABHD12* expression was identified in photoreceptor and bipolar cells according to the Human Protein Atlas. In addition, *ABHD12* was found in microglia located in the outer plexiform layer of the retina. Assuming the same underlying cause for neurodegeneration in the central nervous system and the inner ear, this might also impact the retina and inner ear via microglial regulation (Fiskerstrand et al. 2009; Nishiguchi et al. 2014; Chen et al. 2013). Polyneuropathy, characterized by highly variable clinical manifestations, typically presents between ages 7 and 53. This variability, the most common symptom among patients, is thought to stem from microglial activation and increased neuroinflammation due to phosphatidylserine accumulation.

In this study, genetic testing revealed a compound heterozygous variants in the *ABHD12* gene in the proband. In the family, the proband's father carried a nonsense variant of the *ABHD12* gene. Funduscopic imaging revealed that the macula OCT showed disappearance of the external limiting membrane and the ellipsoidal zone in the fovea centralis, with multiple punctate hyperreflexias visible in the photoreceptor outer segments. Fundus autofluorescence examination revealed punctate hypofluorescence in the fovea centralis. The proband's mother and younger brother carried copy number variants of the *ABHD12* gene, yet neither displayed clinical symptoms. From a molecular perspective, the nonsense variant in the *ABHD12* gene prevented the synthesis of functional protein. However, family members with a heterozygous variant might exhibit mild clinical symptoms and structural abnormalities in the macular region, yet retain normal visual acuity and other visual functions (visual field, and ERG). It was further confirmed that *ABHD12* is a recessive gene, and heterozygous variants do not cause disease or result in a mild clinical phenotype. Only homozygous or compound heterozygous variants can lead to disease. Chen DH (Chen et al. 2013) conducted a quantitative and qualitative protein evaluation of lymphoblastoid cells from a PHARC patient with a homozygous variant, which revealed that the patient was completely devoid of *ABHD12* activity. Her mother, a heterozygous carrier, exhibited 50% *ABHD12* activity without clinical symptoms. This may be attributable to the induced pluripotent stem cells from fibroblasts differentiating into the affected cell line, thereby performing their relevant functions. This research provides new insights into the pathological mechanisms of inherited retinal diseases and offers a hypothesized basis for treatment. The genotypes and phenotypes of compound homozygous variants manifest similarly within the same family but vary significantly across different families. For example, variants c.337_338delGAinsTTT (Asp113Phefs15), c.846_852duptaag (P.IS285fsX1), and c.379_385delaactactinsattcc Ttataccattgtagtcttactgcttttggtga‐acaca (p.Asn127Aspfs23) exhibit the same clinical manifestations. The primary mechanism involves the production of a termination codon, leading to the premature termination of protein translation. Compound heterozygous variants are less prevalent than homozygous variants, and correlating genotype with phenotype proves challenging due to limited clinical data. Therefore, establishing an accurate genotype–phenotype relationship is essential to obtain quantitative data on symptoms at a similar stage, requiring collaborative attention, and evaluation across multiple disciplines. However, implementing this is challenging due to the high variability in the manifestations of neurological, hearing, and visual impairments among patients. Moreover, initial medical consultations usually concentrate on localized lesions, which frequently results in misdiagnoses. Several previous patients, including the current one under our consideration, were initially diagnosed with conditions like deafness, RP, or Usher syndrome. Nevertheless, a comprehensive diagnosis demands genetic testing. The *ABHD12* gene, with a transcript size of 1.1 Kb (NM—001042472.3), is a promising candidate for adenoviral—vector—based gene therapy. Further basic and clinical research on PHARC syndrome will provide a solid foundation for future gene therapy endeavors.

## 5 Conclusion

We report on a patient with PHARC syndrome from a Chinese family, who harbors a compound heterozygous variant (comprising a recognized nonsense variant and a novel copy number variant) within the *ABHD12* gene. Our research expands the spectrum of pathogenic variants and phenotypes associated with *ABHD12*—PHARC syndrome. Moreover, our findings underline the significance of genetic testing, which serves as a valuable tool for confirming the diagnosis of PHARC syndrome and can promote early intervention and long‐term follow‐up evaluation of this disease.

## Author Contributions

Meijiao Ma and Weining Rong wrote the main manuscript, Jinhai Ma collected cases data and Yuanyuan Lian followed up patients. Weining Rong and Xunlun Sheng polished the article. All authors read and approved the final manuscript.

## Ethics Statement

The human studies were approved by the Ethics Committee of Gansu Aier Ophthalmology Hospital, adhering to local legislation and institutional requirements.

## Consent

All participants provided written informed consent. Consent for publication of potentially identifiable images or data was obtained from the participants or, for minors, their legal guardians.

## Conflicts of Interest

The authors declare no conflicts of interest.

## Data Availability

The data that support the findings of this study are openly available in the GenBank repository (https://www.ncbi.nlm.nih.gov/genbank/). Accession numbers: ID: (SCV005077801, SCV005077802).
